# ERICA: prevalences of hypertension and obesity in Brazilian adolescents

**DOI:** 10.1590/S01518-8787.2016050006685

**Published:** 2016-02-02

**Authors:** Katia Vergetti Bloch, Carlos Henrique Klein, Moyses Szklo, Maria Cristina C Kuschnir, Gabriela de Azevedo Abreu, Laura Augusta Barufaldi, Gloria Valeria da Veiga, Beatriz Schaan, Thiago Luiz Nogueira da Silva, Maurício Teixeira Leite de Vasconcellos, and other authors

**Affiliations:** I Instituto de estudos em Saúde Coletiva. Universidade Federal do Rio de Janeiro. Rio de Janeiro, RJ, Brasil; II Departamento de Epidemiologia e Métodos Quantitativos em Saúde. Escola Nacional de Saúde Pública Sérgio Arouca. Fundação Oswaldo Cruz. Rio de Janeiro, RJ, Brasil; III Faculdade de Ciência Médicas. Núcleo de Estudos da Saúde do Adolescente. Universidade do Estado do Rio de Janeiro. Rio de Janeiro, RJ, Brasil; IV Programa de Pós-Graduação em Saúde Coletiva. Instituto de Medicina Social. Universidade do Estado do Rio de Janeiro. Rio de Janeiro, RJ, Brasil; V Departamento de Vigilância de Doenças e Agravos Não Transmissíveis e Promoção da Saúde. Secretaria de Vigilância em Saúde. Ministério da Saúde. Brasília, DF, Brasil; VI Instituto de Nutrição Josué de Castro. Universidade Federal do Rio de Janeiro. Rio de Janeiro, RJ, Brasil; VII Departamento de Medicina Interna. Faculdade de Medicina. Universidade Federal do Rio Grande do Sul. Porto Alegre, RS, Brasil; VIII Serviço de Endocrinologia. Hospital de Clínicas de Porto Alegre. Porto Alegre, RS, Brasil; IX Instituto de Estudos em Saúde Coletiva. Universidade Federal do Rio de Janeiro. Rio de Janeiro, RJ, Brasil; X Escola Nacional de Ciências Estatísticas. Fundação Instituto Brasileiro de Geografia e Estatística. Rio de Janeiro, RJ, Brasil

**Keywords:** Adolescent, Obesity, epidemiology, Hypertension, epidemiology, Cross-Sectional Studies

## Abstract

**OBJECTIVE:**

To estimate the prevalence of arterial hypertension and obesity and the population attributable fraction of hypertension that is due to obesity in Brazilian adolescents.

**METHODS:**

Data from participants in the Brazilian Study of Cardiovascular Risks in Adolescents (ERICA), which was the first national school-based, cross-section study performed in Brazil were evaluated. The sample was divided into 32 geographical strata and clusters from 32 schools and classes, with regional and national representation. Obesity was classified using the body mass index according to age and sex. Arterial hypertension was defined when the average systolic or diastolic blood pressure was greater than or equal to the 95^th^ percentile of the reference curve. Prevalences and 95% confidence intervals (95%CI) of arterial hypertension and obesity, both on a national basis and in the macro-regions of Brazil, were estimated by sex and age group, as were the fractions of hypertension attributable to obesity in the population.

**RESULTS:**

We evaluated 73,399 students, 55.4% female, with an average age of 14.7 years (SD = 1.6). The prevalence of hypertension was 9.6% (95%CI 9.0-10.3); with the lowest being in the North, 8.4% (95%CI 7.7-9.2) and Northeast regions, 8.4% (95%CI 7.6-9.2), and the highest being in the South, 12.5% (95%CI 11.0-14.2). The prevalence of obesity was 8.4% (95%CI 7.9-8.9), which was lower in the North region and higher in the South region. The prevalences of arterial hypertension and obesity were higher in males. Obese adolescents presented a higher prevalence of hypertension, 28.4% (95%CI 25.5-31.2), than overweight adolescents, 15.4% (95%CI 17.0-13.8), or eutrophic adolescents, 6.3% (95%CI 5.6-7.0). The fraction of hypertension attributable to obesity was 17.8%.

**CONCLUSIONS:**

ERICA was the first nationally representative Brazilian study providing prevalence estimates of hypertension in adolescents. Regional and sex differences were observed. The study indicates that the control of obesity would lower the prevalence of hypertension among Brazilian adolescents by 1/5.

## INTRODUCTION

Hypertension (HT) is an important public health problem in Brazil^21^ and worldwide, contributing substantially for mortality due to cardiovascular diseases^3^. Genetic, environmental and behavioral factors interact in the genesis of HT, with excessive weight being among them. Excessive weight is often associated to HT, not only as a causal factor, but also due to its interaction with other factors related to the two conditions^24^. In 2011, the prevalence of self-reported HT in Brazilian adults was 22.7%, which had been stable between 2006 and 2011^1^. On the other hand, the prevalence of excess weight was estimated at 49.0% in the 2008-2009 *Pesquisa de Orçamento Familiar* (POF – Brazilian Household Budget Survey)^a^. The significant increase in both sexes recorded by the POF over the last 20-30 indicates that in less than 10 years about two-thirds of Brazilian adults may be overweight. In adolescents, overweight increased six times in males and almost three times in females, reaching a prevalence of about 20.0% in both sexes in 2008-2009^a^.

Studies performed in various countries have observed an increase in the prevalence of HT in adolescents and a concomitant increase in obesity rates^9^ ^,^ ^20^ ^,^ ^26^. However, during a systematic review, including 122,053 adolescents, which evaluated over 55 studies of the five continents, Moraes et al.^13^ noted a reduction of the prevalence of hypertension in more recent studies, despite the increasing prevalence of obesity.

Freedman et al.^6^ analyzed secular trends in blood pressure during a longitudinal study with children and also observed that blood pressure levels did not follow increases in body mass index (BMI). The authors suggest that other factors have an influence on blood pressure, such as early childhood nutrition and increased birth weight, which offset the effect of obesity in this study, as well as in others. However, there has been an observed association between excess weight or obesity with HT in adolescents^5^ ^,^ ^11^ ^,^ ^18^.

In a systematic review of studies that evaluated adolescents between 10 and 20 years of age, predominantly from the Southeast and Northeast regions of Brazil, an overall HT prevalence of 8.1% was found, (95%CI 6.2-10.5), along with great variability between studies. This variability can be attributed to the great variation in the methods used to measure and classify HT, which indicates the need to standardize methods to measure blood pressure to increase the accuracy of the measurements and allowing comparability among results^10^.

The objective of this study was to estimate the prevalence of HT and obesity as well as the population attributable fraction (PAF) of HT that results from obesity in adolescents.

## METHODS

The Brazilian Study of Cardiovascular Risks in Adolescents (ERICA) is a national school-based study. During this study, adolescents aged between 12 and 17 years, which represents the age corresponding to the concept of adolescence as adopted by the *Estatuto da Criança e do Adolescente* (Child and Adolescent Statute)^b^ were selected. Adolescents with no temporary or permanent physical disabilities, who studied in the last three grades of elementary school or in the first three grades of high school at public and private schools, in cities with over 100,000 inhabitants, from all the five Brazilian macro-regions, were evaluated.

The research population was stratified into 32 geographical strata, which consisted of the 27 state capitals and five strata of the other municipalities with more than 100,000 inhabitants (medium- and large-sized) from each of the five Brazilian macro-regions. Thus, the sample was representative for medium- and large-sized cities at a national and regional level and for each state capital. A detailed description of the sample can be seen in Vasconcellos et al.^25^

During ERICA, data were collected by a team of previously trained evaluators using standard techniques. Three questionnaires were applied (one for the adolescents, one for the parents and one for the school). Anthropometric and blood pressure evaluation was conducted, while a 24-hour food intake record was taken along with laboratory blood tests. The questionnaire for the adolescent was self-filled using an electronic data collector, the personal digital assistant (PDA).

The anthropometric data measured were: height, weight, and waist and arm circumferences. Weight was evaluated using a P200M Líder^®^ scale, which has a capacity of up to 200 kg and a 50 g variation. Height was measured using a portable and collapsable Alturaexata^®^ stadiometer, with a 1 mm resolution and measuring capacity of up to 213 cm. The specific procedures for each measurement are described in detail in Bloch et al.^4^

Obesity was classified according to BMI, namely the body mass (kg) divided by the square of the body height (m). To classify the nutritional status of the adolescents, reference curves from the World Health Organization (WHO) were adopted^17^,using the BMI-for-age chart, according to sex. The following cut-off points were adopted: Z-score < -3 (very low weight); Z-score ≥ -3 and < -2 (low weight); Z-score ≥ -2 and ≤ 1 (eutrophy); score-Z > 1 and ≤ 2 (overweight); Z-score > 2 (obesity).

Blood pressure was verified using a digital monitor (Omron 705-IT), which had been validated for use with adolescents^23^, and was taken using the adolescent’s right arm, with the subject sitting with their feet on the ground, and with an appropriately sized cuff for the size of the arm, all performed according to recommendations from the literature^15^. Three consecutive measurements for each individual were performed, with an interval of three minutes between them. The first measurement was disregarded, and the mean of the last two measurements were considered^4^.

The adolescents were classified as: normotensive, if systolic and diastolic blood pressure were lower than the values of the 90^th^ percentile for height, sex and age; prehypertensive (PH), if the systolic or diastolic blood pressure were between the 90^th^ and 95^th^ percentiles or systolic blood pressure greater than or equal to 120 mmHg or diastolic blood pressure greater than or equal to 80 mmHg, but with a percentile below 95; and, hypertensive, if the systolic or diastolic blood pressure corresponded to the 95^th^ percentile or higher^15^.

A procedure manual, in addition to training videos related to blood pressure and anthropometric evaluation, were developed, to ensure the procedures were standardized and to minimize measurement errors. A pre-test of the research instruments and procedures was performed at a school in Rio de Janeiro. In addition, a pilot study was carried out in 2012 at 15 schools, three in each of the five participating cities (Rio de Janeiro, Cuiaba, Feira de Santana, Campinas and Botucatu)^2^.

The field team was trained and certified before beginning the study, and it was systematically reviewed. The measurements were subject to quality control throughout the data collection period (blood pressure, weight, height, and waist and arm circumference). The information was regularly analyzed to search for trends and patterns that could result in problems in the procedures performed by the interviewers, technicians or data processors.

The distribution of the sample’s coverage was analyzed according to sex, age (information obtained from the adolescent’s questionnaire and confirmed using records supplied by the schools), and Brazilian macro-region. The prevalences and 95% confidence intervals (95%CI) of prehypertension, HT, excess weight and obesity were estimated, as were the prevalence of HT in obese and non-obese adolescents, which was done according to sex and age group (12 to 17 and 14 to 15 years of age), for Brazil as a whole, by capital, for the medium- and large-sized municipalities in the countryside of the regions and for each macro-region.

To estimate the prevalences and their 95%CI, subroutines for complex samples from the software Stata^c^, version 14 were used. In addition to the natural weights of the design, post-stratification estimators were used to modify the natural weights by calibration factors. These calibration factors were obtained by dividing the total population by the estimated total for the natural weights by the post-strata, defined as 12 estimation areas, which correspond to combinations of age and sex, considering the population data of adolescents enrolled in all schools, projected for December 31, 2013. Population estimates for the domains were obtained by processing micro-data from the 2000 and 2010 IBGE Population Censuses^d^. Natural weights were calculated in such a way that the participants represent non-participants and, subsequently, the calibration corrected the joint distributions of sex, age and sampling stratum, according to the estimates of these areas in the population. Therefore, the losses do not lead to errors in the estimates, only in precision losses^25^.

The differences between the subgroups were analyzed based on the 95%CI. The fractions attributable to obesity for HT in the population attributable fraction (PAF) were estimated as a percentage, based on the following equation:

*PAF* = (*p*ht(pop) - *p*ht(nob)) / *p*ht(pop) × 100

In which,

*p*_ht (pop)_ = estimated overall prevalence of HT, for all adolescents;

*p*_ht (nob)_ = estimated prevalence of HT in the non-obese.

ERICA was approved by the Research Ethics Committee (CEP) from each of the 27 participating institutions, one in each state of the Brazilian Federation, and from the three institutions that participated only in the pilot study. We included only adolescents who agreed to participate in the study by signing an assent form and who brought an informed consent form signed by their legal guardian, when required by the local CEP (Universidade Federal de Mato Grosso do Sul, Universidade Federal de Goiás, Universidade Federal da Bahia, Universidade Federal de Roraima and Secretaria Estadual de Educação de Minas Gerais).

## RESULTS

We obtained complete data on the anthropometry and blood pressure of 73,399 students, from a total of 102,327 eligible, aged between 12 and 17 years, according to the schools’ registration systems. Therefore, the overall coverage of medium- and large-sized municipalities in Brazil was 71.7%, ranging from 56.8% male adolescents, aged between 15 and 17 years, from the Midwest region, to 83.6% female adolescents, aged between 12 to 14 years, from the South region. The national coverage of eligible female adolescents was 74.8% (40,675), the coverage of eligible male adolescents was 68.3% (32,724). The study did not considered as eligible 215 pregnant adolescents, 171 female, and 193 male adolescents due to physical disabilities that rendered their measuring unfeasible, which represented 0.6% of the total number of adolescents aged from 12 to 17 years. More male adolescents refused to participate in the study than females in all age groups and in all regions. The number of younger participants was always greater than that of older participants, in both sexes and in all regions. The greatest coverage among the regions was in the South, while the smallest was in the Midwest region, in both sexes and in all age groups. The remaining regions (North, Northeast and South) had similar coverage levels.

The mean age of all the adolescents included in the sample was 14.7 years old (SD = 1.6), while the percentage of older adolescents aged between 15 and 17 years was 54.1%. Table 1 presents the absolute and relative frequencies of the observed scholars and the corresponding estimated population, by sex, age and macro-region.

**Table 1:** Scholars from the ERICA sample and the estimated population according to the IBGE, according to sex, age group and macro-regions in municipalities with more than 100,000 inhabitants. ERICA, Brazil, 2013-2014.

Sex and Age	Brazil	%	North	%	Northeast	%	Midwest	%	Southeast	%	South	%
Observed in the ERICA sample												
Female	40,675	55.4	8,159	11.1	12,691	17.3	5,556	7.6	9,326	12.7	4,943	6.7
12-14 years old	18,497	25.2	3,722	5.1	5,781	7.9	2,519	3.4	4,278	5.8	2,197	3.0
15-17 years old	22,178	30.2	4,437	6.0	6,910	9.4	3,037	4.1	5,048	6.9	2,746	3.7
Male	32,724	44.6	6,808	9.3	10,096	13.8	4,016	5.5	7,457	10.2	4,347	5.9
12-14 years old	15,178	20.7	3,147	4.3	4,566	6.2	1,930	2.6	3,523	4.8	2,012	2.7
15-17 years old	17,546	23.9	3,661	5.0	5,530	7.5	2,086	2.8	3,934	5.4	2,335	3.2

Total	73,399	100	14,967	20.4	22,787	31.0	9,572	13.0	16,783	22.9	9,290	12.7

Estimated population (IBGE)												
Female	5,052,137	49.8	427,997	4.2	1,082,851	10.7	388,843	3.8	2,557,985	25.2	594,461	5.9
12-14 years old	2,650,761	26.1	225,587	2.2	562,205	5.5	202,923	2.0	1,344,038	13.2	316,008	3.1
15-17 years old	2,401,376	23.7	202,410	2.0	520,646	5.1	185,920	1.8	1,213,947	12.0	278,453	2.7
Male	5,095,563	50.2	427,365	4.2	1,082,182	10.7	389,167	3.8	2,595,521	25.6	601,328	5.9
12-14 years old	2,697,440	26.6	224,876	2.2	569,111	5.6	204,588	2.0	1,375,262	13.6	323,603	3.2
15-17 years old	2,398,123	23.6	202,489	2.0	513,071	5.1	184,579	1.8	1,220,259	12.0	277,725	2.7

Total	10,147,700	100	855,362	8.4	2,165,033	21.3	778,010	7.7	5,153,506	50.8	1,195,789	11.8

Population estimate source: http://www.ibge.gov.br/home/estatistica/populacao/projecao_da_populacao/2013/default.shtm

IBGE: Brazilian Institute of Geography and Statistics

Throughout Brazil as a whole and in all its regions, both the prevalences of PH and HT were greater in male adolescent (Table 2). The prevalence of HT was highest in male adolescent aged between 15 and 17 years than in those aged between 12 and 14 in all regions, despite the difference having only been significant in the North region; for PH, the same trend was significant in all regions. In female adolescents, the prevalence of HT was highest among the younger individuals in the North, Northeast and Southeast regions, despite only being significant in the Northeast region. In the Midwest and South regions, the prevalences among older females were slightly larger, albeit not significantly. The prevalence of PH among older female adolescents was larger in all regions, but only with a significant difference in the Southeast region.

**Table 2:** Prevalence (%) and 95%CI of prehypertension and hypertension in medium- and large-sized municipalities for Brazil and macro-regions, according to sex and age group. ERICA, 2013-2014.

Sex/Age	Brazil		North		Northeast		Midwest		Southeast		South	
					
	%	95%CI	%	95%CI	%	95%CI	%	95%CI	%	95%CI	%	95%CI
Prehypertension												
Female	8.9	8.1-9.7	8.4	7.7-9.2	8.8	8.0-9.8	8.7	7.5-10.0	8.3	7.2-9.7	11.6	8.7-15.4
12-14 years old	7.8	6.9-8.7	8.0	6.8-9.3	8.4	7.1-9.8	8.4	7.1-9.9	6.5	5.5-7.8	11.5	7.4-17.3
15-17 years old	10.1	8.9-11.4	8.9	7.8-10.2	9.4	8.1-10.8	9.0	7.4-11.0	10.4	8.3-12.9	11.8	9.7-14.3
Male	20.0	18.9-21.1	18.0	16.6-19.4	19.1	17.5-21.0	22.1	20.4-24.0	19.8	18.0-21.8	22.3	20.7-24.0
12-14 years old	11.7	10.8-12.7	9.6	8.3-11.1	12.1	10.4-14.0	14.0	12.3-16.0	11.3	9.9-12.9	12.4	9.8-15.5
15-17 years old	29.3	27.4-31.4	27.3	25.0-29.7	26.9	23.6-30.5	31.1	28.2-34.3	29.3	25.9-33.1	33.9	31.5-36.5

Total	14.5	13.8-15.1	13.2	12.4-14.1	14.0	12.9-15.1	15.4	14.3-16.6	14.1	13.0-15.3	17.0	15.6-18.5

Hypertension												
Female	7.3	6.6-8.2	6.2	5.4-7.1	6.2	5.4-7.0	6.1	5.1-7.2	7.8	6.5-9.3	9.2	7.5-11.3
12-14 years old	7.8	6.9-8.7	7.2	6.1-8.5	7.4	6.3-8.7	5.9	4.5-7.8	8.0	6.5-9.7	9.0	7.0-11.6
15-17 years old	6.9	5.8-8.1	5.1	4.1-6.2	4.9	4.1-5.8	6.2	4.9-7.9	7.5	5.7-10.0	9.4	7.3-12.1
Male	11.9	11.1-12.8	10.7	9.5-11.9	10.5	9.1-12.2	11.3	10.0-12.8	11.9	10.5-13.4	15.8	14.0-17.8
12-14 years old	10.9	10.0-12.0	8.5	7.3-9.9	9.1	7.4-11.2	9.8	8.0-11.9	11.6	10.1-13.4	13.7	11.4-16.3
15-17 years old	13.0	11.5-14.6	13.1	11.5-14.8	12.1	10.1-14.4	13.1	10.8-15.7	12.2	9.7-15.2	12.2	15.0-21.9

Total	9.6	9.0-10.3	8.4	7.7-9.2	8.4	7.6-9.2	8.7	7.9-9.6	9.8	8.8-11.0	12.5	11.0-14.2

About two-thirds of the adolescents attended class during the morning, and the rest during the afternoon. Each student was evaluated during their class time. The prevalence of HT in adolescents studying in the morning session and the afternoon session were compared for Brazil and the macro-regions; no statistically significant differences were observed according to the 95%CI analysis (Table 3).

**Table 3:** Prevalence (%) of hypertension according to study shift, for both Brazil and the macro-regions. ERICA, 2013-2014.

Macro-region	Morning		Afternoon	
	
	%	95%CI	%	95%CI
Brazil	9.9	9.2-10.8	9.0	8.0-10.0
North	8.2	7.5-9.0	8.7	7.4-10.2
Northeast	8.2	7.7-9.4	8.1	6.8-9.8
Midwest	9.5	8.7-10.3	7.0	5.4-9.1
Southeast	9.9	8.7-11.4	9.4	7.7-11.5
South	13.2	11.5-15.1	10.9	8.5-13.9

The prevalences of overweight individuals (Table 4) were similar between sexes, except for older adolescents in the Northeast region, where we observed more overweight female adolescents. Regarding obesity (Table 4), we observed a higher prevalence in male than in female adolescents. There was a tendency in the younger adolescents to have higher prevalences of excess weight and obesity then in older adolescents for both sexes.

**Table 4:** Prevalence (%) and 95%CI of excess weight and obesity in medium- and large-sized municipalities for Brazil and macro-regions, according to sex and age group. ERICA, 2013-2014.

Sex/Age	Brazil		North		Northeast		midwest		Southeast		South	
					
	%	95%CI	%	95%CI	%	95%CI	%	95%CI	%	95%CI	%	95%CI
Overweight												
Female	17.6	16.4-18.9	15.5	14.1-16.9	17.7	16.2-19.2	16.4	15.0-17.8	17.5	15.3-19.9	20.3	18.0-22.9
12-14 years old	19.2	17.5-21.0	17.1	15.1-19.4	20.6	18.0-23.6	18.4	16.4-20.7	18.4	15.5-21.6	21.8	17.7-26.6
15-17 years old	15.9	14.6-17.3	13.6	12.1-15.3	14.4	13.1-15.9	14.1	12.5-15.9	16.5	14.1-19.2	18.7	16.7-20.9
Male	16.6	15.6-17.8	15.1	13.9-16.4	15.9	14.4-17.4	16.1	14.6-17.7	17.2	15.4-19.2	17.0	14.4-19.9
12-14 years old	17.6	15.9-19.3	16.5	14.6-18.7	19.7	17.3-22.5	16.7	14.4-19.2	17.1	14.3-20.3	17.0	13.7-20.9
15-17 years old	15.6	14.1-17.2	13.6	12.1-15.2	11.5	10.2-13.0	15.4	12.9-18.3	17.4	14.7-20.4	17.0	13.6-21.0

Total	17.1	16.3-18.0	15.3	14.4-16.2	16.8	15.6-18.0	16.2	15.1-17.4	17.4	15.8-19.0	18.7	17.4-20

Obese												
Female	7.6	7.1-8.3	5.6	4.9-6.4	6.8	6.0-7.6	5.9	5.3-6.6	8.1	7.1-9.2	9.8	8.1-11.7
12-14 years old	8.5	7.7-9.4	6.4	5.3-7.7	7.4	6.3-8.6	6.2	5.2-7.3	8.9	7.6-10.3	12.1	9.5-15.4
15-17 years old	6.7	5.8-7.7	4.7	4.0-5.5	6.1	5.1-7.2	5.6	4.7-6.7	7.3	5.7-9.3	7.1	5.5-9.0
Male	9.2	8.4-9.9	7.6	6.6-8.8	8.1	6.7-9.9	8.9	7.4-10.6	9.1	8-.010.4	12.4	11-13.9
12-14 years old	10.8	9.7-12.0	7.9	6.7-9.3	9.4	7.3-12.0	11.4	8.9-14.6	10.7	9-.012.6	15.6	13.2-18.3
15-17 years old	7.3	6.6-8.1	7.3	5.9-9.0	6.8	5.6-8.1	6.1	4.8-7.7	7.4	6.2-8.8	8.7	7.2-10.5

Total	8.4	7.9-8.9	6.6	6.0-7.2	7.4	6.5-8.5	7.4	6.6-8.3	8.6	7.8-9.5	11.1	10.0-12.3

The highest prevalences of HT (Table 2) and obesity (Table 4) occurred in the South region, which differed significantly from all other regions except in female adolescents from the Southeast region.

**Figure 1:**
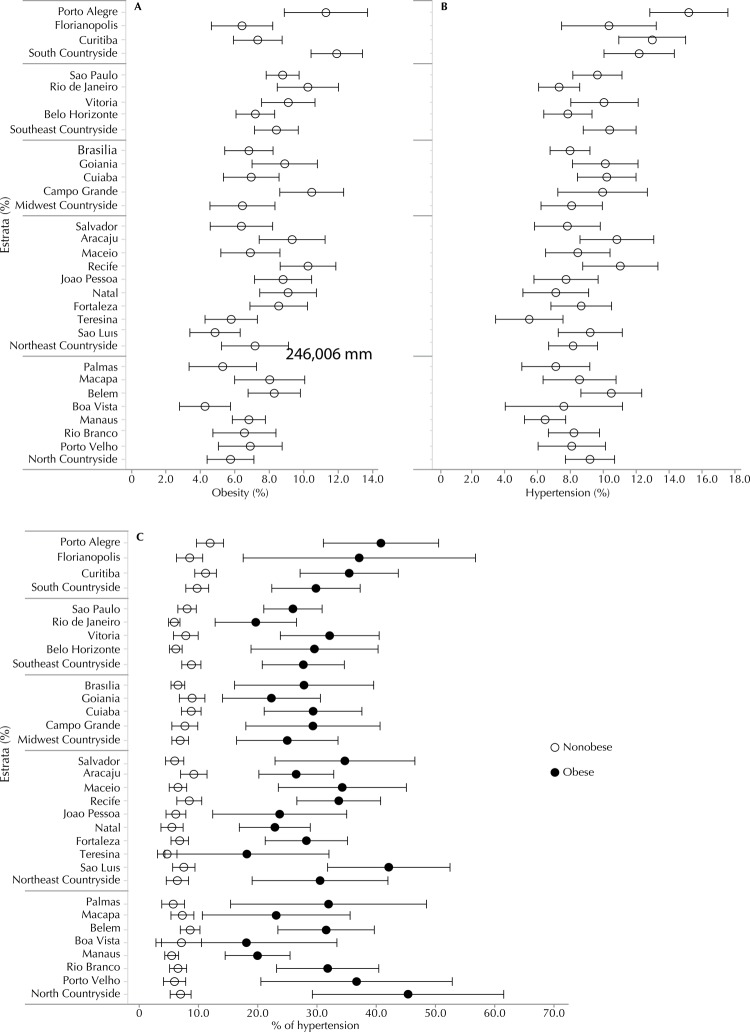
Prevalence (%) of obesity (1A), hypertension (1B) and prevalence of hypertension in obese and nonobese adolescents (1C) by strata of the capitals and Brazil’s countryside* macro-regions. ERICA, 2013-2014.

Figure 1 shows the prevalences of obesity (1A) and HT (1B) in the Brazilian state capitals and the strata from the countryside areas of the regions. Porto Alegre was the capital that had the highest prevalence of HT and obesity. The lowest prevalences of obesity were observed in Boa Vista, Sao Luis, and Palmas, and the lowest prevalences of HT in Teresina, Manaus, Natal, and Palmas. Despite the prevalences of HT and obesity having tended to follow a pattern, this was not always the case. Rio de Janeiro and Natal had relatively low prevalences of HT despite having presented relatively high prevalences of obesity. The opposite was observed in Sao Luis, Florianopolis, Cuiaba and Curitiba.

* Countryside refers to the medium- and large-sized municipalities, except the capitals of each macro-region.

Figure 1(C) shows the prevalences of HT in obese and nonobese adolescents by strata (capitals and countryside of each macro-region). Only in Teresina, PI, and in Boa Vista, RR, were the prevalences of HT in obese and nonobese adolescents not significantly different. In all the other 25 state capitals and in the strata of each countryside region, the prevalence of obese adolescents was significantly higher than nonobese. In the stratum of the interior of the North region and in Porto Velho, the prevalence of HT in obese adolescents was more than six times greater than nonobese. Even in the strata in which this difference was smaller, such as in Boa Vista, Goiania and Aracaju, the prevalence of HT in obese adolescents was 2.5 to 3 times greater than in the nonobese.

**Figure 2:**
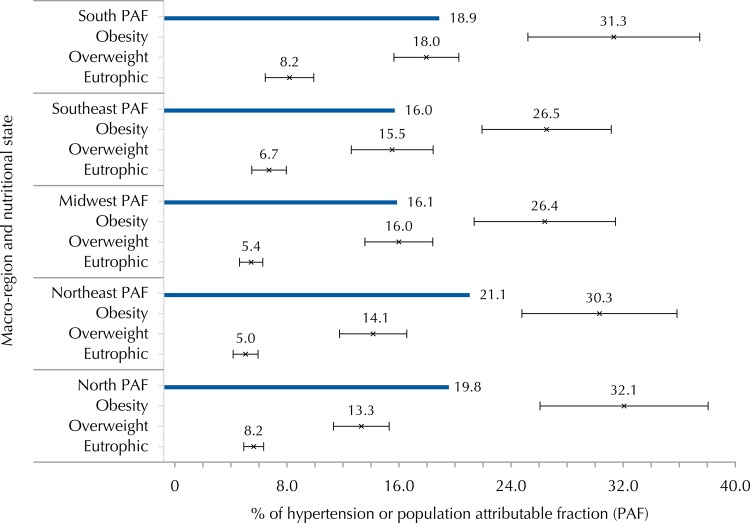
Prevalence (%) of hypertension, 95%CI and FAP (%) according to nutritional status per macro-region. ERICA, Brazil, 2013-2014.

We observed a growing trend in the prevalence of HT from the normal weight (eutrophic) to obese category (Figure 2). Of the total prevalence of HT in adolescents, 17.8% can be attributed to obesity in Brazil. The prevalence of HT attributed to obesity was higher in the North and Northeast regions, where the prevalence in obese was almost six times higher than in nonobese (Figure 2).

PAF: Population attributable fraction of obesity (v*ersus* nonobese) for hypertension

## DISCUSSION

ERICA was the first Brazilian study to estimate HT prevalence in adolescents with national and regional representation. This study’s results showed that 24.0% of Brazilian adolescents who attend schools in municipalities with more than 100,000 inhabitants have high blood pressure (pre-hypertension or hypertension) and 25.0% are overweight. Male adolescents have higher prevalences of HT and obesity, and, despite the prevalence of HT being greater among older adolescents (especially in males), obesity is higher in both male and female young adolescents. The South region had the highest prevalence of HT and obesity. The prevalence of HT was always greater among obese teenagers than among those with normal weight.

Approximately 30.0% of the data set analyzed in this study refused to participate in this study. Refusals were more common in states that required an informed consent form to be signed by a legal guardian, with two of these states being in the Midwest, one in the Northeast and one in the North region (data not shown).

Since there were a greater proportion of non-participants from male sex, public schools, and they were a little older, calculating the variances of the estimates; to verify if they are greater or lesser, is impossible. This is because two types of errors would have to be considered, the first being the sampling error, by the modification of the natural weights, and the non-sampling error, by response losses from non-participants (the variation of the participants prevalences could be greater or lesser), with undetermined directions. It is unlikely that the causes for the losses in this population of young people are related to the problems explored in this study (hypertension and obesity), as they are to factors related to class attendance (less in public schools and in males), and the refusal of the schools to allow pre-entrance exam students to participate (older adolescents), it is plausible to assume that the impact of the non-responses in the estimates presented was small.

About of 10.0% of adolescents observed in ERICA were classified as hypertensive. This prevalence was slightly higher than what was observed in other studies performed in Brazil. During a meta-analysis, Magliano et al.^10^ observed a 8.1% prevalence of hypertension (95%CI 6.2-10.5), which followed the same sex pattern found in this study, with it being higher in male adolescents, 8.8% (95%CI 5.8-13.0), than female ones, 6.3% (95%CI 4.4-9.0). Studies in Brazilian municipalities in different regions found results that were similar to those found in ERICA. Cuiaba, MT, Midwest region, had a prevalence of 11.0% in female adolescents and 12.8% in male adolescents, with a prevalence ratio of 1.17 (95%CI 0.90-1.60)^14^. Belem, PA, North region, had a prevalence of 8.0% and 6.5% in male and female adolescents, respectively (OR = 0.89; 95%CI 0.59-1.33)^19^. In Porto Alegre, in the South region of Brazil, Schommer et al.^22^, found a HT prevalence of 11.3% and a PH prevalence of 16.2% (95%CI 10.2-22.1) in adolescents of both sexes.

During a meta-analysis with studies from various countries, Moraes et al.^13^ found prevalences of HT a little higher than those in ERICA: 11.2% for the general population, with higher prevalences in male (13.0%) than in female (9.6%) adolescents. In 14 Latin America studies that were included in this meta-analysis, the combined prevalence was lower, 6.2% (95%CI 3.1-10.6). The values observed in ERICA were also higher than those found in North American countries and the in Middle East, but lower than those in European, Asian and African countries, which were also included in this meta-analysis. Among the possible explanations for the sexes differences, the authors of the meta-analysis discuss the role of intra-abdominal fat accumulation, which is higher in male adolescents. This accumulation leads to increased sympathetic activity, which in turn increases sodium reabsorption, thereby causing increased peripheral vascular resistance and, consequently, higher blood pressure. The presence of this intra-abdominal fat also leads to an increase in pro-inflammatory cytokines^24^, which may contribute to increased blood pressure values. The increase of plasma testosterone levels during this period of sexual maturation may also contribute to this difference. On the other hand, eating habits and healthy behaviors are influenced by several factors, and these associations differ according to sex^12^.

Regarding overweight and obesity, the prevalences observed in ERICA were lower for overweight and higher for obesity than those observed in the 2008-2009 POF (20.5% and 4.9%, respectively)^a^. Results from Schommer et al.^22^, in Porto Alegre, RS, namely a 17.8% (95%CI 14.7-21.4) prevalence for overweight and 9.8% (95%CI 7.5-12.7) for obesity, were similar to those observed in ERICA in the South region of Brazil. In the South macro-region, both the HT and obesity prevalences were the highest, followed by the Southeast, and the lowest were in the North and Northeast regions. Analyses of other varying characteristics among the regions, such as food consumption and physical activity frequency may assist in the understanding of this distribution.

ERICA results showed that almost one-fifth of the HT prevalence in adolescents in Brazil schools can be attributed to obesity. In absolute figures, about 200,000 Brazilian adolescents would not have HT if they were not obese. Despite the study having simultaneously evaluated both blood pressure and body mass index (BMI), due to the young age of the population under investigation, with no clinical sign of cardiovascular or renal disease yet, it is possible to assume that excess weight precedes an increase in blood pressure. In this scenario, eliminating obesity would significantly reduce the number of hypertensive adolescents, as well as decrease the number of adults with cardiovascular or renal diseases in the future. A study in Belem, PA^19^, did not observe any association between HT and obesity, while in Salvador, BA^7^, and in Porto Alegre, RS^22^, a correlation between anthropometric indicators and blood pressure was observed.

Data from 3,383 American adolescents of the National Health and Nutrition Examination Survey (NHANES ) showed prevalence gradients of prehypertension and HT among normal weight, excess weight and obese categories^11^. Chorin et al.^5^ analyzed data from 714,922 adolescents, taken between 1998 and 2011 during their admission to the Israeli armed forces, and found an association between BMI and systolic and diastolic blood pressure, while observing a trend of increased BMI during the period analysed. Raj et al.^18^ studied around 12,000 children aged between five and 16 years in southern India, from 2003 to 2004 and 2005 to 2006, and noted that blood pressure levels followed the increasing BMI trend, which was higher in rural areas, in public schools and in females.

The Bogalusa study followed children and adolescents for 28 years on average. While doing so they observed that the left ventricular mass of the heart in adults aged between 24 and 46 years presented an adverse effect from excess adiposity and high blood pressure early in childhood, which shows that high blood pressure and adiposity in childhood increases the cardiovascular risk during adulthood^8^.

In ERICA, blood pressure was measured only in one occasion using an oscillometric monitor. The IV^th^ Report on the Diagnosis, Evaluation, and Treatment of High Blood Pressure in Children and Adolescents^15^ recommends that the blood pressure in children and adolescents be preferably measured using the auscultation method. However, the Report recognizes the simplistic nature of the oscillometric method, as well as its ability to minimizes observer bias, which are fundamental issues in population studies. The report also recommends that high blood pressure must be measured and confirmed on separate occasions due to the regression to the mean phenomenon. Therefore, it is possible that the prevalences observed in this study overestimate the prevalence observed while measuring more than once, however, they are comparable to the majority of epidemiological studies, which measured blood pressure on a single occasion due to logistical and cost reasons.

The results show that, despite being required to consider the role that other factors have on the early phases (e.g., socioeconomic factors), reducing obesity can substantially reduce the prevalence of HT in adolescents, which can substantially reduce cardiovascular risks during adulthood. The attributable fraction estimate should be carefully interpreted due to possible confounding variables. However, the consistency in the positive association between HT and obesity, found in each of the 32 strata of this sample, is worth noting. Future analyses of ERICA, considering possible confounding factors and effect modifiers of this relationship at an individual level, could provide more precise estimates.

The increased incidence of obesity can have an impact on life expectancy reducing its growing tendency^16^. Understanding the relationship between obesity and socioeconomic and behavioral characteristics can be useful to develop more effective strategies to prevent obesity in young people, which will reduce the complications from such, including hypertension, and not only ensure that life expectancy continues to grow, but also improve the quality of life for future generations.

## Footnotes

a Instituto Brasileiro de Geografia e Estatística. Pesquisa de Orçamentos Familiares (POF): antropometria e estado nutricional de crianças, adolescentes e adultos no Brasil, 2008-2009. Rio de Janeiro (RJ); 2010 [cited 2015 Nov 7]. Available from: http://www.abeso.org.br/uploads/downloads/70/553a23f27da68.pdf

b Brasil. Lei nº 8.069, de 13 de julho de 1990. Dispõe sobre o Estatuto da Criança e do Adolescente e dá outras providências. Brasília (DF); 1990 [cited 2015 Nov 7]. Available from: http://www.planalto.gov.br/ccivil_03/leis/L8069.htm

c Stata Corp. Stata: statistical software for professionals. Version 14.0. College Station, Texas; 2015.

d Instituto Brasileiro de Geografia e Estatística. Projeção da população do Brasil por sexo e idade: 2000-2060: projeção da população das Unidades da Federação por sexo e idade: 2000-2030. Rio de Janeiro (RJ); 2013 [cited 2015 Sept 4]. Available from: http://www.ibge.gov.br/home/estatistica/populacao/projecao_da_populacao/2013/default.shtm
