# Supplementary material for: ERICA: prevalences of hypertension and obesity in Brazilian adolescents
Source: Rev Saude Publica. 2016 Feb 2;50(Suppl 1):9s. doi: 10.1590/S01518-8787.2016050006685 (PMC4767032; doi:10.1590/S01518-8787.2016050006685)
Supplement: Translation

# Translation: ERICA: prevalências de hipertensão arterial e obesidade em adolescentes brasileiros

**Authors:** Katia Vergetti Bloch, Carlos Henrique Klein, Moyses Szklo, Maria Cristina C Kuschnir, Gabriela de Azevedo Abreu, Laura Augusta Barufaldi, Gloria Valeria da Veiga, Beatriz Schaan, Thiago Luiz Nogueira da Silva, Maurício Teixeira Leite de Vasconcellos, e demais autores

**Affiliations:** I Instituto de estudos em Saúde Coletiva. Universidade Federal do Rio de Janeiro. Rio de Janeiro, RJ, Brasil; II Departamento de Epidemiologia e Métodos Quantitativos em Saúde. Escola Nacional de Saúde Pública Sérgio Arouca. Fundação Oswaldo Cruz. Rio de Janeiro, RJ, Brasil; III Faculdade de Ciência Médicas. Núcleo de Estudos da Saúde do Adolescente. Universidade do Estado do Rio de Janeiro. Rio de Janeiro, RJ, Brasil; IV Programa de Pós-Graduação em Saúde Coletiva. Instituto de Medicina Social. Universidade do Estado do Rio de Janeiro. Rio de Janeiro, RJ, Brasil; V Departamento de Vigilância de Doenças e Agravos Não Transmissíveis e Promoção da Saúde. Secretaria de Vigilância em Saúde. Ministério da Saúde. Brasília, DF, Brasil; VI Instituto de Nutrição Josué de Castro. Universidade Federal do Rio de Janeiro. Rio de Janeiro, RJ, Brasil; VII Departamento de Medicina Interna. Faculdade de Medicina. Universidade Federal do Rio Grande do Sul. Porto Alegre, RS, Brasil; VIII Serviço de Endocrinologia. Hospital de Clínicas de Porto Alegre. Porto Alegre, RS, Brasil; IX Instituto de Estudos em Saúde Coletiva. Universidade Federal do Rio de Janeiro. Rio de Janeiro, RJ, Brasil; X Escola Nacional de Ciências Estatísticas. Fundação Instituto Brasileiro de Geografia e Estatística. Rio de Janeiro, RJ, Brasil

**Keywords:** Adolescente, Obesidade, epidemiologia, Hipertensão, epidemiologia, Prevalência, Estudo Transversal

## Abstract

**OBJETIVO:**

Estimar as prevalências de hipertensão arterial e obesidade e a fração atribuível populacional de hipertensão arterial devida à obesidade em adolescentes brasileiros.

**MÉTODOS:**

Foram avaliados dados dos participantes do Estudo de Riscos Cardiovasculares em Adolescentes, estudo seccional l nacional de base escolar. A amostra foi dividida em 32 estratos geográficos e conglomerados de escolas e turmas, com representatividade nacional, macrorregional e de capitais. Obesidade foi classificada pelo índice de massa corporal segundo idade e sexo. Considerou-se hipertensão arterial a média da pressão arterial sistólica ou diastólica maior ou igual ao percentil 95 da curva de referência. Foram estimadas prevalências e intervalos de confiança de 95% (IC95%) de hipertensão arterial e de obesidade, nacionais e nas macrorregiões do País, por sexo e grupo etário, assim como as frações de hipertensão atribuíveis à obesidade na população.

**RESULTADOS:**

Foram avaliados 73.399 estudantes, 55,4% do sexo feminino, com média de idade 14,7 anos (DP = 1,6). A prevalência de hipertensão arterial foi 9,6% (IC95% 9,0-10,3); sendo as mais baixas observadas nas regiões Norte, 8,4% (IC95% 7,7-9,2) e Nordeste, 8,4% (IC95% 7,6-9,2) e a mais alta na região Sul, 12,5% (IC95% 11,0-14,2). A prevalência de obesidade foi 8,4% (IC95% 7,9-8,9), mais baixa na região Norte e mais alta na Sul. As prevalências de hipertensão arterial e obesidade foram maiores no sexo masculino. Adolescentes com obesidade tiveram prevalência de hipertensão arterial mais elevada, 28,4% (IC95% 25,5-31,2), do que aqueles com sobrepeso, 15,4% (IC95% 13,8-17,0), ou eutróficos, 6,3% (IC95% 5,6-7,0). A fração de hipertensão arterial atribuível à obesidade foi de 17,8%.

**CONCLUSÕES:**

O ERICA foi o primeiro estudo brasileiro com representatividade nacional a estimar a prevalência de hipertensão arterial aferida em adolescentes. A fração da prevalência de hipertensão arterial atribuível à obesidade mostrou que cerca de 1/5 dos hipertensos poderiam não ser hipertensos se não fossem obesos.

## INTRODUÇÃO

A hipertensão arterial (HA) é um importante problema de saúde pública no Brasil^21^ e no mundo, contribuindo de forma relevante para a mortalidade por doenças cardiovasculares^3^.Fatores genéticos, ambientais e comportamentais interagem na gênese da HA, figurando dentre eles o excesso de peso. Esse, por sua vez, frequentemente se associa à HA não apenas como fator causal, mas pela interação com outros fatores compartilhados pelas duas condições^24^. A prevalência de HA autorreferida em adultos brasileiros foi de 22,7% em 2011 e vinha se mantendo estável entre 2006 e 2011^1^. A prevalência de excesso de peso, por outro lado, foi estimada em 49,0% na Pesquisa de Orçamento Familiar (POF) de 2008-2009^a^. O aumento importante em ambos os sexos nos últimos 20-30 anos registrado pela POF indica que em menos de 10 anos cerca de dois terços dos adultos brasileiros poderão ter excesso de peso. Em adolescentes, o excesso de peso aumentou seis vezes no sexo masculino e quase três vezes no feminino, alcançando em 2008-2009 prevalência de cerca de 20,0% em ambos os sexos^a^.

Estudos em diferentes países observaram aumento da prevalência de HA em adolescentes e concomitante aumento da obesidade^9^ ^,^ ^20^ ^,^ ^26^. Entretanto, em revisão sistemática que incluiu 122.053 adolescentes avaliados em 55 estudos dos cinco continentes, Moraes et al.^13^ observaram redução da prevalência de hipertensão nos estudos mais recentes, apesar do aumento da prevalência de obesidade.

Freedman et al.^6^ analisaram a tendência secular da pressão arterial em um estudo longitudinal com crianças e também observaram que os níveis tensionais não acompanharam o aumento do índice de massa corporal (IMC). Os autores sugerem que outros fatores que influenciam a pressão arterial, tais como alimentação na primeira infância e aumento do peso ao nascer, tenham contrabalançado o efeito da obesidade nesse estudo, assim como em outros. Todavia, a associação entre excesso de peso ou obesidade com HA em adolescentes tem sido observada^5^ ^,^ ^11^ ^,^ ^18^.

No Brasil, revisão sistemática de estudos que avaliaram adolescentes de 10 a 20 anos, predominantemente das regiões Sudeste e Nordeste, encontrou prevalência global de HA de 8,1%, (IC95% 6,2-10,5), com grande variabilidade entre os estudos. Essa variabilidade pode ser atribuída à grande variação dos métodos de aferição e classificação de HA utilizados, indicando a necessidade de padronização dos métodos de aferição da pressão arterial para aumentar a acurácia das medidas e possibilitar a comparabilidade dos resultados^10^.

O objetivo do presente estudo foi estimar as prevalências de HA e de obesidade e a fração atribuível populacional (FAP) de HA devido à obesidade em adolescentes brasileiros.

## MÉTODOS

O Estudo de Riscos Cardiovasculares em Adolescentes (ERICA) é um estudo seccional, nacional, de base escolar. Foram selecionados adolescentes de 12 a 17 anos de idade, que corresponde ao conceito de adolescência adotado pelo Estatuto da Criança e do Adolescente^b^. Foram avaliados adolescentes sem deficiência física provisória ou permanente, que estudavam nas três últimas séries do ensino fundamental ou nas três séries do ensino médio em escolas públicas e privadas de municípios com mais de 100 mil habitantes, de todas as cinco macrorregiões do Brasil.

A população de pesquisa foi estratificada em 32 estratos geográficos, constituídos pelas 27 capitais e por cinco conjuntos dos demais municípios com mais de 100 mil habitantes (médio e grande porte) de cada uma das cinco macrorregiões do País. Assim, a amostra tem representatividade para o conjunto de municípios de médio e grande porte no nível nacional e regional e para cada capital. A descrição pormenorizada da amostra encontra-se em Vasconcellos et al.^25^

A coleta de dados do ERICA foi realizada por equipe de avaliadores previamente treinados utilizando técnicas padronizadas. Foram aplicados três questionários (um para os adolescentes, um para os pais, e outro para a escola). Foi realizada avaliação antropométrica e de pressão arterial, aplicado o recordatório alimentar de 24 horas e realizados exames laboratoriais de sangue. O questionário do adolescente foi autopreenchido usando-se um coletor eletrônico de dados, o *personnal digital assistant* (PDA).

As medidas antropométricas aferidas foram estatura, peso, circunferências da cintura e do braço. O peso foi obtido em balança eletrônica Líder^®^ com capacidade de 200 kg e variação de 50 g. A estatura foi medida utilizando-se estadiômetro portátil e desmontável da marca Alturexata^®^, com resolução de 1 mm e campo de uso de até 213 cm. Os procedimentos específicos para cada medida estão descritos detalhadamente em Bloch et al.^4^

Obesidade foi classificada segundo o IMC, razão entre o peso (kg) e o quadrado da estatura (m). Para a classificação do estado nutricional dos adolescentes, foram adotadas as curvas de referência da Organização Mundial de Saúde (OMS)^17^, utilizando como índice o IMC-para-idade, segundo sexo. Os pontos de corte adotados foram: escore-Z < -3 (muito baixo peso); escore-Z ≥ -3 e < -2 (baixo peso); escore-Z ≥ -2 e ≤ 1 (eutrofia); escore-Z > 1 (sobrepeso) e ≤ 2; escore-Z > 2 (obesidade).

A pressão arterial foi verificada com monitor digital (Omron 705-IT), validado para uso em adolescentes^23^, no braço direito, com o aluno sentado e com os pés no chão, e com manguito de tamanho apropriado para o tamanho do braço seguindo as recomendações da literatura^15^.Realizou-se três medidas consecutivas para cada indivíduo, com intervalo de três minutos entre elas. A primeira medida foi descartada e utilizou-se a média das duas últimas medidas^4^.

Os adolescentes foram classificados como: normotensos, se a pressão arterial sistólica e a diastólica eram mais baixas que os valores do percentil 90 para sua estatura, sexo e idade; pré-hipertensos (PH), se a pressão sistólica ou a diastólica encontravam-se entre os percentis 90 e 95 ou com a pressão arterial sistólica maior ou igual a 120 mmHg ou a diastólica maior ou igual a 80 mmHg, mas com percentil menor que 95; e, hipertensos, se a pressão arterial sistólica ou a diastólica correspondia ao percentil 95 ou superior^15^.

Foram elaborados manual de procedimentos e vídeos para treinamento referente à avaliação antropométrica e de pressão arterial, para garantir a padronização dos procedimentos e minimizar erros de aferição. Foi realizado pré-teste dos procedimentos e instrumentos da pesquisa em uma escola do Rio de Janeiro e um estudo piloto em 2012 em 15 escolas, três em cada uma das cinco cidades participantes (Rio de Janeiro, Cuiabá, Feira de Santana, Campinas e Botucatu)^2^.

A equipe de campo foi treinada e certificada antes do início do estudo e reavaliada sistematicamente. Durante toda a coleta de dados realizou-se controle de qualidade das aferições (pressão arterial, peso, estatura, circunferência da cintura e do braço). As informações foram analisadas regularmente na busca de tendências e padrões que pudessem resultar em problemas nos procedimentos realizados, seja por entrevistadores, técnicos ou processadores de informação.

A distribuição da cobertura da amostra foi analisada por sexo, idade (informações obtidas no questionário do adolescente e confirmadas no cadastro fornecido pelas escolas) e macrorregião brasileira. Foram estimadas prevalências e intervalos de confiança de 95% (IC95%) de pré-hipertensão, HA, sobrepeso e obesidade, assim como a prevalência de HA em adolescentes obesos e não obesos, por sexo e grupo etário (12 a 14 e 15 a 17 anos), para todo o Brasil, por capitais, para o conjunto de municípios de médio e grande porte do interior das regiões e para cada macrorregião.

Para as estimações de prevalências e seus IC95% utilizaram-se rotinas para amostras complexas do programa Stata^c^, versão 14. Além dos pesos naturais do desenho, foram utilizados estimadores de pós-estratificação que modificaram os pesos naturais por fatores de calibração. Esses fatores de calibração foram obtidos dividindo-se os totais populacionais pelos totais estimados pelos pesos naturais para os pós-estratos, definidos como 12 domínios de estimação, correspondentes às combinações de idade e sexo, considerando os dados populacionais de adolescentes matriculados em todas as escolas, projetados para 31/12/2013. As estimativas de população para os domínios foram obtidas por meio de processamento dos microdados dos Censos Demográficos de 2000 e 2010 do IBGE^d^. Os pesos naturais foram calculados de forma que os participantes representassem os não participantes e, posteriormente, a calibração corrigiu as distribuições conjuntas de sexo, idade e estrato amostral, de acordo com as estimativas desses domínios na população. Portanto, as perdas não levam a erros nas estimativas pontuais, apenas em perdas de precisão^25^.

As diferenças entre subgrupos foram analisadas com base nos IC95%. Foram estimadas as frações atribuíveis à obesidade para HA na população (FAP), em percentual, com base na seguinte equação:

*PAF* = (*p*ht(pop) - *p*ht(nob)) / *p*ht(pop) × 100

Onde,

*p*_ha(pop)_ = prevalência estimada de HA global, de todos os adolescentes

*p*_ha(nob)_ = prevalência estimada de HA em não obesos

O ERICA foi aprovado pelo Comitê de Ética em Pesquisa (CEP) de cada uma das 27 instituições participantes, uma em cada unidade da federação brasileira, e das três instituições que participaram do estudo piloto. Só foram incluídos no estudo os adolescentes que concordaram em participar do estudo assinando o termo de assentimento e trouxeram termo de consentimento livre e esclarecido assinado por responsável, quando exigido por CEP locais (Universidade Federal de Mato Grosso do Sul, Universidade Federal de Goiás, Universidade Federal da Bahia, Universidade Federal de Roraima e Secretaria Estadual de Educação de Minas Gerais).

## RESULTADOS

Foram obtidos dados completos de antropometria e pressão arterial de 73.399 estudantes de um total de 102.327 elegíveis na faixa etária de 12 a 17 anos, de acordo com o cadastro das escolas. Portanto, a cobertura global dos municípios de médio e grande porte do País foi de 71,7%, variando de 56,8% dos adolescentes do sexo masculino de 15 a 17 anos da região Centro-Oeste a 83,6% das adolescentes do sexo feminino de 12 a 14 anos da região Sul. A cobertura nacional dos adolescentes elegíveis do sexo feminino foi de 74,8% (40.675) e dos adolescentes elegíveis do sexo masculino foi de 68,3% (32.724). Não foram considerados elegíveis 215 adolescentes grávidas, e 171 adolescentes do sexo feminino e 193 do sexo masculino por apresentarem deficiência física que inviabilizava as medidas, representando 0,6% do total de adolescentes de 12 a 17 anos. A recusa de participação no estudo foi maior nos adolescentes do sexo masculino do que no feminino em todas as faixas etárias e em todas as regiões. A participação dos mais jovens foi sempre maior do que a dos mais velhos, em ambos os sexos e em todas as regiões. A maior cobertura entre as regiões foi a da região Sul e a menor a da região Centro-Oeste, em ambos os sexos e grupos etários. As demais regiões, Norte, Nordeste e Sudeste, apresentaram níveis de cobertura semelhantes.

A média de idade de todos os adolescentes na amostra foi de 14,7 anos (DP = 1,6), e o percentual de adolescentes mais velhos, de 15 a 17 anos, foi de 54,1%. A Tabela 1 apresenta as frequências absolutas e relativas de escolares observados e da população correspondente estimada, por sexo idade e macrorregiões.

**Tabela 1:** Escolares da amostra do ERICA e da população estimada segundo o Instituto Brasileiro de Geografia e Estatística, por sexo, grupo etário e macrorregiões em municípios com mais de 100 mil habitantes. ERICA, Brasil, 2013-2014.

Sexo e Idade	Brasil	%	Norte	%	Nordeste	%	Centro-Oeste	%	Sudeste	%	Sul	%
Observados na amostra ERICA												
Feminino	40.675	55,4	8.159	11,1	12.691	17,3	5.556	7,6	9.326	12,7	4.943	6,7
12-14 anos	18.497	25,2	3.722	5,1	5.781	7,9	2.519	3,4	4.278	5,8	2.197	3,0
15-17 anos	22.178	30,2	4.437	6,0	6.910	9,4	3.037	4,1	5.048	6,9	2.746	3,7
Masculino	32.724	44,6	6.808	9,3	10.096	13,8	4.016	5,5	7.457	10,2	4.347	5,9
12-14 anos	15.178	20,7	3.147	4,3	4.566	6,2	1.930	2,6	3.523	4,8	2.012	2,7
15-17 anos	17.546	23,9	3.661	5,0	5.530	7,5	2.086	2,8	3.934	5,4	2.335	3,2

Total	73.399	100	14.967	20,4	22.787	31,0	9.572	13,0	16.783	22,9	9.290	12,7

População estimada (IBGE)												
Feminino	5.052.137	49,8	427.997	4,2	1.082.851	10,7	388.843	3,8	2.557.985	25,2	594.461	5,9
12-14 anos	2.650.761	26,1	225.587	2,2	562.205	5,5	202.923	2,0	1.344.038	13,2	316.008	3,1
15-17 anos	2.401.376	23,7	202.410	2,0	520.646	5,1	185.920	1,8	1.213.947	12,0	278.453	2,7
Masculino	5.095.563	50,2	427.365	4,2	1.082.182	10,7	389.167	3,8	2.595.521	25,6	601.328	5,9
12-14 anos	2.697.440	26,6	224.876	2,2	569.111	5,6	204.588	2,0	1.375.262	13,6	323.603	3,2
15-17 anos	2.398.123	23,6	202.489	2,0	513.071	5,1	184.579	1,8	1.220.259	12,0	277.725	2,7

Total	10.147.700	100	855.362	8,4	2.165.033	21,3	778.010	7,7	5.153.506	50,8	1.195.789	11,8

Fonte da estimativa populacional: http://www.ibge.gov.br/home/estatistica/populacao/projecao_da_populacao/2013/default.shtm

IBGE: Instituto Brasileiro de Geografia e Estatística

No conjunto do País e em todas as regiões, tanto as prevalências de PH como de HA foram maiores nos adolescentes do sexo masculino (Tabela 2). A prevalência de HA foi maior nos adolescentes do sexo masculino de 15 a 17 anos do que nos de 12 a 14 anos em todas as regiões, embora a diferença tenha sido significativa apenas na região Norte; para a PH, a mesma tendência foi significativa em todas as regiões. Já nas adolescentes do sexo feminino, as prevalências de HA foram maiores nas mais jovens nas regiões Norte, Nordeste e Sudeste, embora de forma significativa apenas na região Nordeste. No Centro-Oeste e no Sul, as prevalências entre as mais velhas foram discretamente maiores, mas sem diferença significativa. A prevalência de PH entre as adolescentes do sexo feminino com mais idade foi maior em todas as regiões, mas com diferença significativa apenas no SE.

**Tabela 2:** Prevalências e IC95% de pré-hipertensão e de hipertensão nos municípios de médio e grande porte por macrorregião e Brasil, segundo sexo e grupo etário. ERICA, 2013-2014.

Sexo/Idade	Brasil		Norte		Nordeste		Centro-Oeste		Sudeste		Sul	
					
	%	IC95%	%	IC95%	%	IC95%	%	IC95%	%	IC95%	%	IC95%
Pré-hipertensão												
Feminino	8,9	8,1-9,7	8,4	7,7-9,2	8,8	8,0-9,8	8,7	7,5-10,0	8,3	7,2-9,7	11,6	8,7-15,4
12-14 anos	7,8	6,9-8,7	8,0	6,8-9,3	8,4	7,1-9,8	8,4	7,1-9,9	6,5	5,5-7,8	11,5	7,4-17,3
15-17 anos	10,1	8,9-11,4	8,9	7,8-10,2	9,4	8,1-10,8	9,0	7,4-11,0	10,4	8,3-12,9	11,8	9,7-14,3
Masculino	20,0	18,9-21,1	18,0	16,6-19,4	19,1	17,5-21,0	22,1	20,4-24,0	19,8	18,0-21,8	22,3	20,7-24,0
12-14 anos	11,7	10,8-12,7	9,6	8,3-11,1	12,1	10,4-14,0	14,0	12,3-16,0	11,3	9,9-12,9	12,4	9,8-15,5
15-17 anos	29,3	27,4-31,4	27,3	25,0-29,7	26,9	23,6-30,5	31,1	28,2-34,3	29,3	25,9-33,1	33,9	31,5-36,5

Total	14,5	13,8-15,1	13,2	12,4-14,1	14,0	12,9-15,1	15,4	14,3-16,6	14,1	13,0-15,3	17,0	15,6-18,5

Hipertensão												
Feminino	7,3	6,6-8,2	6,2	5,4-7,1	6,2	5,4-7,0	6,1	5,1-7,2	7,8	6,5-9,3	9,2	7,5-11,3
12-14 anos	7,8	6,9-8,7	7,2	6,1-8,5	7,4	6,3-8,7	5,9	4,5-7,8	8,0	6,5-9,7	9,0	7,0-11,6
15-17 anos	6,9	5,8-8,1	5,1	4,1-6,2	4,9	4,1-5,8	6,2	4,9-7,9	7,5	5,7-10,0	9,4	7,3-12,1
Masculino	11,9	11,1-12,8	10,7	9,5-11,9	10,5	9,1-12,2	11,3	10,0-12,8	11,9	10,5-13,4	15,8	14,0-17,8
12-14 anos	10,9	10,0-12,0	8,5	7,3-9,9	9,1	7,4-11,2	9,8	8,0-11,9	11,6	10,1-13,4	13,7	11,4-16,3
15-17 anos	13,0	11,5-14,6	13,1	11,5-14,8	12,1	10,1-14,4	13,1	10,8-15,7	12,2	9,7-15,2	18,2	15,0-21,9

Total	9,6	9,0-10,3	8,4	7,7-9,2	8,4	7,6-9,2	8,7	7,9-9,6	9,8	8,8-11,0	12,5	11,0-14,2

Cerca de dois terços dos adolescentes estudavam no turno da manhã e o restante no turno da tarde. Cada aluno foi avaliado no seu respectivo turno. Foram comparadas as prevalências de HA dos estudantes do turno da manhã e da tarde, Brasil e macrorregiões, e não foram observadas diferenças estatisticamente significativas pela análise dos IC95% (Tabela 3).

**Tabela 3:** Prevalência de hipertensão arterial segundo turno de estudo, para o Brasil e macrorregiões. ERICA, 2013-2014.

Macrorregião	Manhã		Tarde	
	
	%	IC95%	%	IC95%
Brasil	9,9	9,2-10,8	9,0	8,0-10,0
Norte	8,2	7,5-9,0	8,7	7,4-10,2
Nordeste	8,2	7,7-9,4	8,1	6,8-9,8
Centro-Oeste	9,5	8,7-10,3	7,0	5,4-9,1
Sudeste	9,9	8,7-11,4	9,4	7,7-11,5
Sul	13,2	11,5-15,1	10,9	8,5-13,9

As prevalências de sobrepeso (Tabela 4) foram semelhantes entre os sexos, com exceção apenas nos mais velhos do Nordeste, onde foi maior nas adolescentes do sexo feminino. Quanto à obesidade (Tabela 4), verificou-se maior prevalência nos adolescentes do sexo masculino do que nas do sexo feminino. Houve tendência dos adolescentes mais novos apresentarem prevalências de sobrepeso e obesidade maiores do que os mais velhos em ambos os sexos.

**Tabela 4:** Prevalências e IC95% de sobrepeso e de obesidade nos municípios de médio e grande porte por macrorregião e Brasil, segundo sexo e grupo etário. ERICA, 2013-2014.

Sexo/Idade	Brasil		Norte		Nordeste		Centro-Oeste		Sudeste		Sul	
					
	%	IC95%	%	IC95%	%	IC95%	%	IC95%	%	IC95%	%	IC95%
Sobrepeso												
Feminino	17,6	16,4-18,9	15,5	14,1-16,9	17,7	16,2-19,2	16,4	15,0-17,8	17,5	15,3-19,9	20,3	18,0-22,9
12-14 anos	19,2	17,5-21,0	17,1	15,1-19,4	20,6	18,0-23,6	18,4	16,4-20,7	18,4	15,5-21,6	21,8	17,7-26,6
15-17 anos	15,9	14,6-17,3	13,6	12,1-15,3	14,4	13,1-15,9	14,1	12,5-15,9	16,5	14,1-19,2	18,7	16,7-20,9
Masculino	16,6	15,6-17,8	15,1	13,9-16,4	15,9	14,4-17,4	16,1	14,6-17,7	17,2	15,4-19,2	17,0	14,4-19,9
12-14 anos	17,6	15,9-19,3	16,5	14,6-18,7	19,7	17,3-22,5	16,7	14,4-19,2	17,1	14,3-20,3	17,0	13,7-20,9
15-17 anos	15,6	14,1-17,2	13,6	12,1-15,2	11,5	10,2-13,0	15,4	12,9-18,3	17,4	14,7-20,4	17,0	13,6-21,0

Total	17,1	16,3-18,0	15,3	14,4-16,2	16,8	15,6-18,0	16,2	15,1-17,4	17,4	15,8-19,0	18,7	17,4-20

Obesidade												
Feminino	7,6	7,1-8,3	5,6	4,9-6,4	6,8	6,0-7,6	5,9	5,3-6,6	8,1	7,1-9,2	9,8	8,1-11,7
12-14 anos	8,5	7,7-9,4	6,4	5,3-7,7	7,4	6,3-8,6	6,2	5,2-7,3	8,9	7,6-10,3	12,1	9,5-15,4
15-17 anos	6,7	5,8-7,7	4,7	4,0-5,5	6,1	5,1-7,2	5,6	4,7-6,7	7,3	5,7-9,3	7,1	5,5-9,0
Masculino	9,2	8,4-9,9	7,6	6,6-8,8	8,1	6,7-9,9	8,9	7,4-10,6	9,1	8-,010,4	12,4	11-13,9
12-14 anos	10,8	9,7-12,0	7,9	6,7-9,3	9,4	7,3-12,0	11,4	8,9-14,6	10,7	9-,012,6	15,6	13,2-18,3
15-17 anos	7,3	6,6-8,1	7,3	5,9-9,0	6,8	5,6-8,1	6,1	4,8-7,7	7,4	6,2-8,8	8,7	7,2-10,5

Total	8,4	7,9-8,9	6,6	6,0-7,2	7,4	6,5-8,5	7,4	6,6-8,3	8,6	7,8-9,5	11,1	10,0-12,3

As maiores prevalências de HA (Tabela 2) e obesidade (Tabela 4) ocorreram na região Sul, que diferiu significativamente de todas as demais regiões, exceto do Sudeste nas adolescentes do sexo feminino.

A Figura 1 apresenta as prevalências de obesidade (1A) e de HA (1B) nas capitais brasileiras e nos estratos do interior das regiões. Porto Alegre foi a capital com maior prevalência de HA e de obesidade. As prevalências mais baixas de obesidade foram observadas em Boa Vista, São Luís e Palmas e de HA em Teresina, Manaus, Natal e Palmas. Embora as prevalências de HA e de obesidade tendessem a seguir um mesmo padrão, nem sempre isso ocorreu. Rio de Janeiro e Natal apresentaram prevalências de HA relativamente baixas embora tenham apresentado prevalências de obesidade relativamente elevadas. O inverso foi observado em São Luís, Florianópolis, Cuiabá e Curitiba.

**Figura 1:** Prevalências de obesidade (A) e de hipertensão arterial (B) e prevalências de hipertensão em adolescentes sem obesidade e com obesidade (C) por estratos de capital e interior* de macrorregiões do Brasil. ERICA, 2013-2014.

* Interior refere-se aos municípios de médio e grande porte, exceto as capitais de cada macrorregião.

Na Figura 1(C) são apresentadas as prevalências de HA em adolescentes obesos e não obesos por estratos (capitais e interior de cada macrorregião). Apenas em Teresina, PI, e em Boa Vista, RR, as prevalências de HA em adolescentes com obesidade e sem obesidade não foram significativamente diferentes. Em todas as demais 25 capitais e nos estratos do interior de cada região a prevalência dos adolescentes com obesidade foi significantemente maior do que a dos sem obesidade. No estrato do interior da região Norte e em Porto Velho, a prevalência de HA nos adolescentes com obesidade foi mais de seis vezes maior que nos adolescentes sem obesidade. Mesmo nos estratos nos quais essa diferença foi menor, Boa Vista, Goiânia e Aracaju, a prevalência de HA nos adolescentes obesos foi 2,5 a 3 vezes maior que nos não obesos.

Observou-se tendência crescente da prevalência de HA da categoria de peso adequado (eutróficos) à de obesidade (Figura 2). No total do Brasil, 17,8% da prevalência de HA nos adolescentes pode ser atribuída à obesidade. A prevalência de HA atribuída à obesidade foi mais elevada nas regiões Norte e Nordeste, nas quais as prevalências nos obesos foi quase seis vezes maior do que nos não obesos (Figura 2).

**Figura 2:** Prevalência de hipertensão arterial, IC95% e FAP (%) segundo estado nutricional por macrorregião. ERICA, Brasil, 2013-2014.

FAP: Fração atribuível populacional de obesidade (*versus* não obesidade) para hipertensão arterial

## DISCUSSÃO

O ERICA foi o primeiro estudo brasileiro com representatividade nacional e regional a estimar prevalência de HA aferida em adolescentes. Os resultados do estudo mostraram que 24,0% dos adolescentes brasileiros que frequentam escolas em municípios com mais de 100 mil habitantes estão com pressão arterial elevada (pré-hipertensão ou hipertensão) e 25,0% estão com excesso de peso. Os adolescentes do sexo masculino têm maiores prevalências de HA e de obesidade, e, enquanto a prevalência de HA é maior entre os mais velhos (principalmente no sexo masculino), a de obesidade é maior entre os mais novos, em ambos os sexos. A região Sul apresentou as maiores prevalências de HA e de obesidade. A prevalência de HA foi sempre maior entre os adolescentes com obesidade do que entre os com peso adequado.

Para o conjunto de dados analisados nesse estudo, as recusas em participar do estudo foram de aproximadamente 30,0%. Essas recusas foram maiores nos estados que exigiram TCLE assinados pelos responsáveis, sendo dois da região Centro-Oeste, um da Nordeste e um da Norte (dados não apresentados).

Uma vez que os não participantes são em maior proporção do sexo masculino e de escolas públicas, e um pouco mais velhos, não há como calcular os desvios das estimativas, se para mais ou para menos. Isso porque teriam que ser considerados dois tipos de erros, o amostral, pela modificação dos pesos naturais, e o não amostral, pelas perdas de respostas dos não participantes (a variação das prevalências dos participantes poderiam ser para mais ou para menos), com direções indeterminadas. Como é improvável que as causas das perdas nessa população de jovens estejam relacionadas aos problemas em estudo (hipertensão arterial e obesidade), e sim a fatores relacionados à frequência as aulas (menor nas escolas públicas e no sexo masculino) e à recusa das escolas em liberar turmas de pré-vestibular (mais velhos), é plausível supor que o impacto da não-resposta nas estimativas apresentadas foi pequeno.

Cerca de 10,0% dos adolescentes observados no ERICA foram classificados como hipertensos. Essa prevalência foi um pouco maior do que as observadas em outros estudos realizados no Brasil. Em meta-análise, Magliano et al.^10^ observaram prevalência de hipertensão de 8,1% (IC95% 6,2-10,5), seguindo o mesmo padrão por sexo encontrado no presente estudo, maior nos adolescentes do sexo masculino, 8,8% (IC95% 5,8-13,0), do que nas do sexo feminino, 6,3% (IC95% 4,4-9,0). Estudos em municípios brasileiros de diferentes regiões encontraram resultados semelhantes aos do ERICA. Cuiabá, MT, região Centro-Oeste, apresentou prevalência de 11,0% nas adolescentes e 12,8% nos adolescentes, com razão de prevalência de 1,17 (IC95% 0,90-1,60)^14^. Belém, PA, região Norte, apresentou prevalência de 8,0% e 6,5% nos adolescentes e nas adolescentes, respectivamente (OR = 0,89; IC95% 0,59-1,33)^19^. Em Porto Alegre, RS, no Sul do País, Schommer et al.^22^ encontraram prevalências de HA de 11,3% e de PH de 16,2% (IC95% 10,2-22,1) em adolescentes de ambos os sexos.

Em meta-análise com estudos de diversos países, Moraes et al.^13^ encontraram prevalências de HA um pouco mais elevadas do que no ERICA: 11,2% para a população geral, com prevalências maiores nos adolescentes do sexo masculino (13,0%) do que nas do sexo feminino (9,6%). Nos 14 estudos da América Latina incluídos nessa meta-análise, a prevalência combinada foi mais baixa, 6,2% (IC95% 3,1-10,6). Os valores observados no ERICA foram maiores também do que os encontrados em países da América do Norte e do Oriente Médio e menores do que os dos europeus, asiáticos e africanos incluídas nesta mesma meta-análise. Dentre as possíveis explicações para a diferença por sexo, os autores da meta-análise discutem o papel do acúmulo de gordura intra-abdominal, maior nos adolescentes do sexo masculino. Esse acúmulo levaria a uma maior atividade simpática, que por sua vez aumentaria a reabsorção do sódio, causando aumento da resistência vascular periférica e, consequentemente, da pressão arterial. Essa gordura intra-abdominal leva também ao aumento das citocinas pró-inflamatórias^24^, que podem contribuir para o aumento dos valores de pressão arterial. O aumento dos níveis plasmáticos de testosterona nesse período de maturação sexual também pode contribuir para a diferença. Por outro lado, hábitos alimentares e comportamentos saudáveis são influenciados por diversos fatores e essas associações diferem por sexo^12^.

Quanto ao excesso de peso e obesidade, as prevalências observadas no ERICA foram menores para sobrepeso e maiores para obesidade do que as observadas na POF 2008-2009 (20,5% e 4,9%, respectivamente)^a^. Os resultados de Schommer et al.^22^, em Porto Alegre, RS, 17,8% (IC95% 14,7-21,4) de sobrepeso e 9,8% (IC95% 7,5-12,7) de obesidade, foram semelhantes aos da região Sul observados no ERICA. Nesta macrorregião, tanto as prevalências de HA quanto de obesidade foram mais elevadas, seguidas pelas do Sudeste, e foram mais baixas nas regiões Norte e Nordeste. Análises de outras características que variem entre as regiões, tais como consumo alimentar e prática de atividade física, poderão contribuir para a compreensão dessa distribuição.

Os resultados do ERICA mostraram que quase 1/5 da prevalência de HA em adolescentes escolares no Brasil pode ser atribuída à obesidade. Em números absolutos, cerca de 200 mil adolescentes brasileiros não teriam HA se não fossem obesos. Embora o estudo tenha avaliado simultaneamente a pressão arterial e a massa corporal (IMC), por se tratar de uma população jovem, ainda sem manifestações clínicas de doença cardiovascular ou renal, é possível supor que excesso de peso anteceda elevação da pressão arterial. Nesse cenário, a eliminação da obesidade reduziria significativamente o número de adolescentes hipertensos e de futuros adultos com doenças cardiovasculares ou renais. Estudo realizado em Belém, PA^19^, não observou associação entre HA e obesidade, enquanto que em Salvador, BA^7^, e em Porto Alegre, RS^22^, foi observada correlação de indicadores antropométricos com pressão arterial.

Dados de 3.383 adolescentes norte-americanos do *National Health and Nutrition Examination Survey* (NHANES) mostraram gradientes de prevalência de pré-hipertensão e de HA entre as categorias de peso normal, sobrepeso e obesidade^11^. Chorin et al.^5^ analisaram dados de 714.922 adolescentes examinados entre 1998 e 2011 na admissão às forças armadas de Israel e encontraram associação entre IMC e pressão arterial sistólica e diastólica, e observaram tendência de aumento do IMC no período analisado. Raj et al.^18^ estudaram cerca de 12 mil crianças de cinco a 16 anos no Sul da Índia nos períodos de 2003-2004 e 2005-2006 e observaram que os níveis de pressão arterial acompanharam a tendência de aumento do IMC, que foi maior em áreas rurais, em escolas públicas e em meninas.

O estudo Bogalusa acompanhou crianças e adolescentes em média por 28 anos e nele foi observado que a massa do ventrículo esquerdo do coração de adultos com 24 a 46 anos de idade apresentou efeito adverso do excesso de adiposidade e de pressão arterial elevada no início na infância, mostrando que a adiposidade e níveis pressóricos elevados na infância aumentam o risco cardiovascular de adultos^8^.

A pressão arterial no ERICA foi aferida com monitor oscilométrico em uma única ocasião. O *IV* ^*th*^ *Report on the Diagnosis, Evaluation, and Treatment of High Blood Pressure in Children and Adolescents* ^15^ recomenda que a aferição da pressão arterial em crianças e adolescentes seja realizada preferencialmente pelo método auscultatório. Entretanto, reconhece a simplicidade do método oscilométrico, que ainda minimiza o viés de observador, atributos fundamentais em estudos populacionais. Recomenda ainda que pressões elevadas devem ser confirmadas com medidas realizadas em diferentes ocasiões devido ao efeito de regressão à média. Portanto, é possível que as prevalências observadas neste estudo superestimem as prevalências obtidas com medidas em mais de uma ocasião, mas são comparáveis às da maioria dos estudos epidemiológicos, que por razões logísticas e de custo fazem medidas em uma única ocasião.

Os resultados encontrados mostram que, embora o papel de outros fatores que atuam em fases ainda mais precoces (por exemplo, socioeconômicos) devam ser considerados, a redução da obesidade pode reduzir substancialmente a prevalência de HA em adolescentes, diminuindo potencialmente o risco cardiovascular em adultos. A estimativa da fração atribuível deve ser interpretada com cautela devido à possibilidade de confundimento. Todavia, há uma notável consistência na associação positiva entre HA e obesidade que foi encontrada em cada um dos 32 estratos desta amostra. Análises futuras do ERICA que considerem possíveis fatores de confundimento e modificadores de efeito dessa relação no nível individual poderão fornecer estimativas mais precisas de associação.

O aumento da incidência da obesidade pode ter impacto na expectativa de vida a ponto de interromper sua tendência de crescimento^16^. Compreender as relações entre obesidade e diferentes características socioeconômicas e comportamentais pode ajudar na elaboração de estratégias mais eficazes de prevenção da obesidade em jovens, de forma a reduzir suas complicações, entre elas a hipertensão arterial, e garantir não só a tendência de crescimento da expectativa de vida mas também a qualidade de vida das futuras gerações.

## Footnotes

a Instituto Brasileiro de Geografia e Estatística. Pesquisa de Orçamentos Familiares (POF): antropometria e estado nutricional de crianças, adolescentes e adultos no Brasil, 2008-2009. Rio de Janeiro; 2010 [citado 2015 nov 7]. Disponível em: http://www.abeso.org.br/uploads/downloads/70/553a23f27da68.pdf

b Brasil. Lei nº 8.069, de 13 de julho de 1990. Dispõe sobre o Estatuto da Criança e do Adolescente e dá outras providências. Brasília (DF); 1990 [citado 2015 nov 7]. Disponível em: http://www.planalto.gov.br/ccivil_03/leis/L8069.htm

c Stata Corp. Stata: statistical software for professionals. Version 14.0. College Station, Texas; 2015

d Instituto Brasileiro de Geografia e Estatística. Projeção da população do Brasil por sexo e idade: 2000-2060: projeção da população das Unidades da Federação por sexo e idade: 2000-2030. Rio de Janeiro; 2013 [citado 2015 set 4]. Disponível em: http://www.ibge.gov.br/home/estatistica/populacao/projecao_da_populacao/2013/default.shtm
